# Incidental prostate cancer after holmium laser enucleation of the prostate: Critical analysis of independent risk factors and impact on surgical outcomes

**DOI:** 10.1002/bco2.306

**Published:** 2023-10-31

**Authors:** Joao G. Porto, Ruben Blachman‐Braun, Tarek Ajami, Mohamadhusni Zarli, Ryan Chen, Thiago Furtado, Robert Marcovich, Dipen J. Parekh, Hemendra N. Shah

**Affiliations:** ^1^ Desai Sethi Urology Institute, Miller School of Medicine University of Miami Miami Florida USA; ^2^ Dr. Kiran C. Patel College of Osteopathic Medicine Nova Southeastern University Fort Lauderdale Florida USA; ^3^ Faculdade de Ciências Médicas de Minas Gerais Belo Horizonte Brazil

**Keywords:** active surveillance, benign prostatic hyperplasia, holmium laser enucleation of the prostate, incidental prostate cancer, lower tract urinary symptoms

## Abstract

**Objectives:**

The objectives of this study are to evaluate the impact of incidental prostate cancer (iPCa) and its different grade group (GG) on the surgical outcomes of holmium laser enucleation of the prostate (HoLEP) and, furthermore, to assess the independent risk factors associated with the detection of iPCa.

**Patients (or materials) and Methods:**

A retrospective chart review was conducted at a single institution for HoLEP cases that were performed between 2017 and 2022. Patients with a preoperative diagnosis of prostate cancer and those without baseline prostate‐specific antigen (PSA) levels within 1 year were excluded. Four hundred seventeen patients were divided into three groups: benign prostatic hyperplasia—377; clinically insignificant (GG 1)—29; and clinically significant prostate cancer (GG 2–5)—11. The preoperative parameters analysed included age, body mass index, race/ethnicity, use of 5‐alpha‐reductase inhibitors, PSA, prostate size, PSA density, and history of negative prostate biopsy. To evaluate the association between clinical and demographic variables, a multivariable‐adjusted logistic regression analysis was performed. We also assessed intraoperative and post‐operative outcomes among these three groups.

**Results:**

A total of 417 patients were analysed; 40 (9.6%) patients had iPCa, of which 29 (72.5%) and 11 (27.5%) were clinically nonsignificant and significant prostate cancer, respectively. Of all the demographic and preoperative variables analysed, hypertension was significantly associated with overall prostate cancer diagnosis (*p* < 0.05), and no other variable including patient age, preoperative PSA, PSA density, prostate size, or prior prostate biopsy were associated with increased risk of overall prostate cancer or clinically significant prostate cancer diagnosis. Most cases of iPCa were GG1, and 34 (85%) were managed with active surveillance.

**Conclusion:**

The rate of iPCa after HoLEP was 9.6%, with most cases being GG 1 (72.5%) and managed through active surveillance. Age, prostate size, baseline PSA, and prior negative prostate biopsies were not associated with increased risk of iPCa.

## 1 INTRODUCTION

A wide range of treatments are available for benign prostatic hyperplasia (BPH), and procedures involving the removal of prostate tissue can lead to the detection of incidental prostate cancer (iPCa). Autopsy samples have revealed that approximately 40% of men over 60 years old and nearly 60% of men over 80 years old have prostate cancer.^1^, ^2^ Although prostate‐specific antigen (PSA) testing has greatly improved the diagnosis and treatment of prostate cancer, reducing the incidence of iPCa by about 65%, it is important to inform patients about the possibility of this diagnosis after surgical interventions for BPH.^1^

A review conducted by Yilmaz et al. demonstrated a rate of iPCa ranging from 5.64% to 23.3% after holmium laser enucleation of the prostate (HoLEP).^3^ A temporal analysis noted that older patients have an increased incidence of grade group (GG) 1 iPCa that was associated with decrease in rate of preoperative prostate biopsy.^4^ Multiple studies have explored the incidence of iPCa following BPH procedures. However, there are only three, with conflicting findings, which have specifically examined potential predictive factors for iPCa after HoLEP. Bhojani et al. revealed that older age, elevated PSA level, and low resected volume are associated with a higher likelihood of iPCa.^5^ In contrast, Elkousky et al. identified age, PSA, and PSA density as predictive factors for iPCa, while Ohwaki et al. suggested a connection between diabetes and iPCa.^6^, ^7^ Importantly, none of these studies provided information about the number of patients who underwent prostate biopsy prior to HoLEP. Only Elkousky et al. mentioned that 54.3% of patients with Gleason Grade 1 had negative biopsy results before undergoing HoLEP, and the remaining authors did not report the rate of negative biopsies among patients with low‐ and high‐risk prostate cancer.^5^, ^6^, ^7^ Hence, the literature is controversial and inconclusive regarding various factors that are associated with iPCa after HoLEP.

The primary aim of this study was to evaluate various demographic and preoperative factors associated with the risk of iPCa diagnosis, particularly high‐risk prostate cancer after HoLEP. The secondary aims of the study were to investigate the overall incidence of iPCa after HoLEP, assess the potential impacts of iPCa on surgical outcomes of HoLEP, and evaluate the impact of preoperative magnetic resonance imaging (MRI) and prostate biopsy on iPCa. This information would add to our existing knowledge of this relatively infrequent but clinically significant scenario.

## 2 PATIENTS (OR MATERIALS) AND METHODS

We conducted a retrospective chart review of patients who underwent HoLEP with “En Bloc” technique performed by a single surgeon with active involvement of residents and fellows at a single institution from July 2017 to August 2022. Institutional Review Board approval was obtained (20180511). Preoperatively, all patients with elevated PSA prior to HoLEP were further evaluated by PSA velocity (comparing with past PSA results when available), PSA density, 4K score, and/or prostate MRI. In case of suspicious results from these tests, they would undergo prostate biopsy by shared decision‐making. All patients with prostate imaging reporting and data system (PIRADS) 4 or 5 lesions on MRI were advised to undergo MRI‐fusion biopsy of the prostate preoperatively.

Three months post‐operatively, all patients were recommended PSA to establish nadir level following HoLEP. Those diagnosed with iPCa were counselled in depth about the role of prostate MRI, confirmatory prostate biopsy, and/or continued PSA monitoring to assist in further management of iPCa. Those individuals with Gleason 6 iPCa and having nadir PSA ≤ 0.6 ng/mL were usually recommended serial PSA every 6 months. Conversely, those with Gleason 6 iPCa and having nadir PSA ≥ 0.6 ng/mL and those with intermediate‐ or high‐risk iPCa were recommended additional assessment with MRI prostate, confirmatory MRI‐fusion biopsy, computerized tomography scan, and bone scan.

After selection, 501 patients were included, and then, we excluded those with a previous diagnosis of prostate cancer (*n* = 55) or prostate/pelvic radiotherapy (*n* = 1) and those without baseline PSA (*n* = 9) or not recorded radiological estimation of prostate size (*n* = 19). After exclusions, 417 patients were divided into three groups based on their histopathological evaluation obtained after HoLEP: BPH, low‐risk prostate cancer/clinically nonsignificant (GG 1), and intermediate‐ and high‐risk prostate cancer/clinically significant (GG 2–5). We collected preoperative information including age, body mass index, race/ethnicity, chronic diseases, use of 5‐alpha‐reductase inhibitors, PSA, prostate size, PSA density, and history of negative prostate biopsy. Resected weight of prostate tissue and operation time were also assessed. Post‐operatively, we collected information related to voiding parameters and complications related to surgery and management for patients diagnosed with iPCa. Evaluation of International Prostate Symptom Score (IPSS), maximum urinary flow rate (Qmax), and post‐void residual urine at 1 year of follow‐up was done.

### 2.1 Statistical analysis

It was performed with SPSS version 28 software. Means and standard deviations (±*SD*) or medians and interquartile ranges [25th–75th] were calculated according to the data distribution through the assessment of normality test (Kolmogorov–Smirnov and Shapiro–Wilk). A comparison of numerical variables between groups was performed using the Analysis of Variance or Kruskal–Wallis test as required. Categorical variables were analysed with a Chi‐square test. To evaluate the association between clinical and demographic variables with overall iPCa (GG 1–5) and clinically significant prostate cancer (GG 2–5), a multivariable‐adjusted logistic regression analysis was performed. The relevant variables were selected based on prior literature search and supported by authors' experience and expertise. A *p*‐value < 0.05 was considered statistically significant.

## 3 RESULTS

A total of 417 patients were analysed; 40 (9.6%) patients had iPCa, of which 29 (72.5%) had low‐risk prostate cancer (GG 1), while 11 (27.5%) had clinically significant prostate cancer (GG 2–5). There was no statistically significant difference in demographic, clinical, and preoperative biochemical characteristics between groups of patients in accordance with the pathology results after HoLEP except for higher incidence of hypertension in patients with iPCa diagnosis (Table 1). The average PSA level and percentage of patients with PSA > 4 ng/mL and PSA density ≥ 0.15 ng/mL/cc were similar in patients in all three groups. In our entire series, 156 (37.4%) of patients underwent preoperative prostate biopsy. Patients with diagnosis of iPCa had similar rate of preoperative prostate biopsy (44.8%) as compared with those with diagnosis of BPH (36.8%) (*p* = 0.580).

**TABLE 1:** Comparison of clinical, demographic, and biochemical characteristics between groups of patients in accordance with the pathology results after holmium laser enucleation of the prostate.

	Overall 417 (100%)	BPH 377 (90.4%)	Low‐risk PCa 29 (7%)	High‐risk PCa 11 (2.6%)	Overall *p*‐value
Age (years)	69.1 ± 8.5	69 ± 8.4	70 ± 7.7	70.6 ± 11.6	0.682
BMI (kg/m^2^)	28 ± 4.5	27.9 ± 4.5	28.2 ± 4.61	29.5 ± 4.7	0.520
Race/ethnicity					
White or Caucasian (%)	348 (83.5%)	315 (83.6%)	24 (82.8%)	9 (81.8%)	0.932
Black/African American (%)	37 (8.9%)	32 (8.5%)	4 (13.8%)	1 (9.1%)	
Asian (%)	4 (1.0%)	4 (1.1%)	0	0	
Multiracial/not reported (%)	28 (6.7%)	26 (6.9%)	1 (3.4%)	1 (9.1%)	
Hypertension	168 (40.3%)	140 (37.1%)	22 (75.9%)	6 (54.5%)	<0.001
Dyslipidaemia	148 (35.5%)	136 (36.1%)	7 (24.1%)	5 (45.5%)	0.339
Diabetes mellitus	101 (24.2%)	90 (23.9%)	8 (27.6%)	3 (27.3%)	0.878
On 5αRi before surgery	199 (47.7%)	181 (48%)	12 (41.4%)	6 (54.5%)	0.710
PSA (ng/mL)	4.4 [2.2–8.1]	4.4 [2.2–8.2]	3.7 [1.7–7.3]	5.1 [1.3–7.9]	0.590
PSA ≥ 4 ng/mL	218 (52.3%)	200 (53.1%)	12 (41.4%)	6 (54.5%)	0.474
Baseline prostate size (cc)	102 [74–155]	105 [74–157]	96 [77.4–119]	102 [48–151]	0.450
PSAd **(**ng/mL/cc)	0.04 [0.024–0.071]	0.041 [0.024–0.071]	0.035 [0.024–0.069]	0.053 [0.021–0.075]	0.939
PSAd ≥ 0.15 ng/mL/cc	24 (5.8%)	22 (5.8%)	1 (3.4%)	1 (9.1%)	0.773
Prior negative prostate biopsy	156 (37.4%)	138 (36.6%)	13 (44.8%)	5 (45.5%)	0.580
Operative time (min)	150 [109–195.5]	150 [109.5–197]	151 [103.5–188]	145 [109–190]	0.735
Prostate resected volume (cc)	80 [45.9–116.4]	80 [47–115.9]	75.5 [31.2–116.3]	55 [24.9–178.1]	0.727
PSA post‐operation^a^ (ng/mL)	0.40 [0.21–0.78]	0.42 [0.21–0.77]	0.40 [0.30–0.91]	0.27 [0.10–0.35]	0.050
Time at PSA post‐operation (months)	3 [2–3]	3 [2–3]	3 [2–3]	3 [2–5]	0.218

*Note*: Mean ± standard deviation; median [interquartile range 25th to 75th].

Abbreviations: 5αRi, 5‐alpha reductase inhibitor; BMI, body mass index; BPH, benign prostatic hyperplasia; PCa, prostate cancer; PSA, prostate‐specific antigen; PSAd, prostate‐specific antigen density.

^a^ Three hundred forty‐six patients were analysed.

Of 40 patients with iPCa, 34 (85%) were placed on active surveillance (AS), while three (7.5%) patients underwent robotic‐assisted laparoscopic prostatectomy (RALP). The remaining three patients received different treatments, including high‐intensity focal ultrasound, androgen deprivation therapy and abiraterone with prednisone due to metastatic disease, and radiation therapy of the prostate (Table 2).

**TABLE 2:** Grade groups (GG) of incidental prostate cancer (PCa) at the time of holmium laser enucleation of the prostate.

	Overall	Active surveillance^a^	HIFU	RALP	Systemic therapy	Radiation therapy
PCa	40 (100%)	34 (85%)	1 (2.5%)	3 (7.5%)	1 (2.5%)	1 (2.5%)
GG1	29 (72.5%)	28 (82.4%)	0	1 (33.3%)	0	0
GG2	8 (20.0%)	5 (14.7%)	1 (100%)	2 (66.7%)	0	0
GG3^b^	1 (2.5%)	0	0	0	0	1 (100%)
GG4^a^	1 (2.5%)	0	0	0	1 (100%)	0
GG5	1 (2.5%)	1 (2.9%)	0	0	0	0

Abbreviations: HIFU, high‐intensity‐focussed ultrasound; RALP, robot‐assisted laparoscopic prostatectomy.

^a^ Patient was found to have metastatic prostate cancer; he was started on Leuprolide with abiraterone and prednisone.

^b^ Patient decided to establish care in another institution and was referred to androgen deprivation therapy and radiation therapy.

On multivariable‐adjusted logistic regression analysis, neither age, race/ethnicity, preoperative prostate size, PSA level, or prostate biopsy results were associated with increased incidence of iPCa in general (GG 1–5), clinically significant prostate cancer (GG 2–5), or low‐risk prostate cancer (GG 1), in particular. We noted that hypertension was associated with increased risk of detecting iPCa in general (odds ratio = 4.256, 95% confidence interval: 2.022–8.958; *p* < 0.001) and low‐risk prostate cancer (odds ratio = 5.749, 95% confidence interval: 2.332–14.178; *p* < 0.001) but was not associated with increased risk of detection of clinically significant iPCa (Table 3). During follow‐up after HoLEP, the incidence of acute urinary retention was 35 (8.4%), urinary tract infection was 31 (7.4%), bladder neck stenosis was eight (1.9%), and urethral stricture was nine (2.2%). When analysing the incidence of complications among groups, it was observed that the frequency of urethral stricture was significantly higher in the group of patients with low‐risk prostate cancer (10.3%) when compared with the rest of the groups (*p* = 0.007) (Table 4). Furthermore, at 1 year of follow‐up, median IPSS, Qmax, and post‐void residual urine were similar between the groups (*p* > 0.05) (Table 5). Out of 40 patients with iPCa diagnosis in our series, 17 had preoperative MRI done, and three of them showed a PIRADS 4 or 5 lesion. These patients with concerning lesions on scan had a higher frequency of high‐risk prostate cancer (*p* < 0.001) (Table 6).

**TABLE 3:** Multivariable‐adjusted logistic regression analysis to assess the association between clinical and demographic variables and incidental prostate cancer GG 1–5 and clinically significant and clinically no significant prostate cancer at the time of holmium laser enucleation of the prostate.

	Prostate cancer GG 1^a^			Prostate cancer GG 1 to 5			Prostate cancer GG 2 to 5		
	OR	95% CI	*p*‐value	OR	95% CI	*p*‐value	OR	95% CI	*p*‐value
Age (per 1 year)	1.022	0.974–1.073	0.371	1.028	0.987–1.071	0.186	1.036	0.962–1.115	0.350
Race/ethnicity									
White or Caucasian	1			1			1		
Black or African American	1.551	0.468–5.144	0.473	1.374	0.461–4.093	0.568	0.861	0.071–10.411	0.906
Multiracial/not reported	0.627	0.078–5.038	0.661	0.845	0.184–3.887	0.828	1.266	0.140–11.434	0.834
Hypertension									
No	1		<0.001	1		< 0.001	1		0.386
Yes	5.749	2.332–14.178		4.256	2.022–8.958		1.772	0.486–6.451	
Dyslipidaemia									
No	1		0.060	1		0.146	1		0.588
Yes	0.416	0.167–1.037		0.574	0.272–1.213		1.421	0.399–5.065	
Diabetes mellitus									
No	1		0.931	1		0.903	1		0.995
Yes	0.961	0.392–2.360		0.953	0.438–2.072		0.996	0.244–4.056	
Preoperative PSA (per 1 ng/mL)	0.988	0.922–1.058	0.727	1.013	0.988–1.039	0.312	1.028	0.999–1.059	0.061
Preoperative prostate size (per 1 cc)	0.996	0.989–1.003	0.281	0.994	0.988–1.001	0.071	0.99	0.977–1.004	0.163
Prior negative prostate biopsy									
No	1		0.189	1		0.157	1		0.531
Yes	1.776	0.754–4.180		1.682	0.819–3.456		1.521	0.409–5.651	

Abbreviations: CI, confidence interval; GG, grade group; OR, odds ratio; PSA, prostate‐specific antigen.

^a^ Only 406 patients were selected (11 patients with GG 2 to GG 5 were excluded for this model).

**TABLE 4:** Comparison of complication rate, duration of catheter post‐operative, and length of stay between groups of patients according to grade group after holmium laser enucleation of the prostate.

	Overall 417 (100%)	BPH 377 (90.4%)	Low‐risk PCa 29 (7%)	High‐risk PCa 11 (2.6%)	*p*‐value
Acute urinary retention (%)	35 (8.4%)	32 (8.5%)	3 (10.3%)	0	0.561
Urinary tract infection (%)	31 (7.4%)	27 (7.2%)	3 (10.3%)	1 (9.1%)	0.802
Bladder neck stenosis (%)	8 (1.9%)	8 (2.1%)	0	0	0.649
Urethral stricture (%)	9 (2.2%)	6 (1.6%)	3 (10.3%)	0	**0.007**
Blood transfusions (%)	10 (2.4%)	9 (2.4%)	0	1 (9.1%)	0.244
Catheterization period (days)	1 [1–1]	1 [1–1]	1 [1–1]	1 [1–1]	0.589
Length of stay (days)	1 [1–1]	1 [1–1]	1 [1–1]	1 [1–1]	0.942

Abbreviations: BPH, benign prostatic hyperplasia; PCa, prostate cancer.

*Note*: Item in bold indicates statistically significant result (*p*‐value < 0.05).

**TABLE 5:** Comparison of IPSS, Qmax, and PVR at 1 year of follow‐up between groups of patients according to grade group after holmium laser enucleation of the prostate.

	Overall	BPH	Low‐risk PCa	High‐risk PCa	Overall *p*‐value
IPSS^a^	0 [0–3]	1 [0–3]	0 [0–1.5]	5 [0–8]	0.129
Qmax^b^	19 [12.7–29.7]	18.8 [12.7–30]	19.7 [12.7–22.9]	20^d^	0.625
PVR^c^	15 [0–38]	15 [0–38]	16 [3–36]	13^d^	0.961

*Note*: Median [interquartile range 25th to 75th].

Abbreviations: BPH, benign prostatic hyperplasia; IPSS, International Prostate Symptom Score; PCa, prostate cancer; PVR, post‐void residual volume; Qmax, maximum urinary flow rate.

^a^ One hundred forty‐two patients were analysed.

^b^ One hundred seventy‐six patients were analysed.

^c^ One hundred eighty‐five patients were analysed.

^d^ Only median is reported due to the limited number of patients.

**TABLE 6:** Comparison of the preoperative characteristics and clinical outcomes in accordance with the preoperative MRI results.

	Not performed *n* = 400 (95.9%)	No suspicious lesion *n* = 14 (3.4%)	PIRADS 4 or 5 *n* = 3 (0.7%)	*p*‐value
Prior biopsy				
No (%)	256 (64%)	4 (28.6%)	1 (33.3%)	**0** **.015**
Yes (%)	144 (36%)	10 (71.4%)	2 (66.7%)	
Preoperative PSA (ng/mL)	4.29 [2.15–8.05]	4.70 [3.13–8.40]	6.21^a^	0.294
PSA density (ng/mL/cc)	0.04 [0.02–0.07]	0.06 [0.03–0.08]	0.07^a^	0.282
Prostate cancer outcome				
BPH (%)	377 (94.3%)	0	0	**< 0.001**
Low‐risk PCa (%)	18 (4.5%)	11 (78.6%)	0	
High‐risk PCa (%)	5 (1.3%)	3 (21.4%)	3 (100%)	

*Note*: Median [interquartile range 25th to 75th]. Items in bold indicate statistically significant results.

Abbreviations: BPH, benign prostatic hyperplasia; PCa, prostate cancer; PIRAD, prostate imaging reporting and data system; PSA, prostate‐specific antigen.

^a^ Only median was able to be calculated.

## 4 DISCUSSION

Despite routine screening efforts, a substantial number of prostate cancer cases in men remain undetected.^8^ As a result, BPH procedures can inadvertently lead to the discovery of malignancy. In our series, we identified iPCa in 9.6% of cases, with 72.5% as GG1 and 27.5% GG 2–5 prostate cancer. The variability observed in the literature regarding incidence of iPCa after endoscopic enucleation can be attributed to the diverse approaches in preoperative evaluation of patients who are potential candidates for surgery.^9^ One of the key aspects influencing this variability is the threshold for performing preoperative prostate biopsy and MRI, which can effectively diagnose a significant proportion of patients with prostate cancer prior to the procedure. Lee et al. proposed an algorithm recommending prostate biopsy for all patients with elevated PSA, regardless of MRI results, and centres adopting this approach are expected to have lower iPCa rates compared with those suggesting biopsy only for suspicious MRI cases.^10^ Previous studies have shown that a negative MRI can also miss 8–20% of clinically significant prostate cancers,^11^, ^12^ while prostate biopsy can miss 30% of prostate cancer cases.^13^ In the present study, 20% patients with negative preoperative MRI and prostate biopsy were found to have clinically significant prostate cancer, and all patients with PIRADS 4 and 5 lesion in the MRI were found to have clinically significant prostate cancer.

Numerous parameters have been historically investigated as potential definitive predictive factors for iPCa. However, previous publications have yielded inconclusive findings, with distinct factors related to this diagnosis being identified. Among the parameters currently receiving more support in the literature are elevated age, baseline PSA, and PSA density.^5^, ^6^, ^9^, ^14^, ^15^, ^16^, ^17^, ^18^ Additionally, other factors such as PSA velocity, prostate size, and decreased resected volume have also been associated with iPCa.^5^, ^6^, ^9^, ^14^, ^15^, ^16^ A previous study comparing patients who had BPH with glands bigger than 100 cc found no association between PSA and PSA density related to iPCa.^19^ In our study, we did not observe an association between these factors and probability of diagnosing iPCa after HoLEP. Nonetheless, we did discover a significant association between hypertension and GG 1 prostate cancer. Other authors have also reported a similar association between prostate cancer and hypertension, although the precise cause‐and‐effect relationship of these findings remains unclear.^20^ A prior study conducted by Ohwaki et al. reported a link between diabetes and the diagnosis of high‐risk iPCa.^7^ However, we did not notice any such association in our study.

Furthermore, we did not observe any significant differences in post‐HoLEP outcomes among patients with BPH, low‐risk, and high‐risk prostate cancer. Post‐operative improvement in voiding parameters was similar in all three groups. Regarding post‐operative complications, we did not find any difference except for the frequency of urethral stricture (10.3%), which was significantly higher in the group of patients with low‐risk prostate cancer (*p* = 0.007). Although we cannot explain this finding, it is important to highlight that we did not do subgroup analysis to look for other factors that might be responsible for increased incidence of stricture formation in this group, and this will be the subject of further research.

Prostate cancer predominantly occurs in the peripheral zone, but managing iPCa after BPH procedures presents a challenge. This stems from the possible coexistence of malignancy in the transition zone.^21^ While it might seem logical to consider prostate biopsy as a solution, past research has shown that post‐transurethral resection of the prostate biopsies do not typically yield further insights into Gleason scores for the majority of cases.^22^ All the patients with low‐risk prostate cancer in our study were managed with AS, except one patient who had high volume disease and underwent RALP, based on his personal desire to receive definitive treatment. Han et al. conducted a study to investigate the management of patients with iPCa and found that nearly 80% of patients were placed under AS. The remaining 20% received immediate treatment, with the decision being significantly influenced by abnormal post‐procedure MRI and staging.^17^ Gellhaus et al. reported safe and promising oncologic results for patients who undergo RALP after HoLEP.^23^ Throughout our study period, we did not observe any patients who discontinued AS, with an average follow‐up of 12 months.

In this study, 11 patients were diagnosed with clinically significant prostate cancer, and their treatment was customized based on their GG, clinical characteristics, and stage (Table 2). One patient with obstructive urinary symptoms despite combined medical management, normal preoperative PSA, and no signs of metastatic disease (bone pain, weight loss, or fatigue) was incidentally diagnosed with prostate cancer GG 4, and after further investigation, he was promptly diagnosed with metastasis, leading to the initiation of androgen deprivation therapy in conjunction to abiraterone with prednisone. Another patient with GG 3 opted for radiation therapy, which has been deemed safe for patients who have undergone HoLEP treatment previously.^24^ We also had a patient with GG 5 managed through AS. This patient was a 90‐year‐old male with a bothersome urinary symptom (IPSS of 25) and recurrent gross haematuria despite combined medical management and prostate artery embolization 1 year back. He had PSA of 3.5 ng/dL and had two negative prostate biopsies in the past 2 years. His MRI performed 4 months prior to HoLEP revealed a cystic cavity in prostate secondary to prior prostate artery embolization. He had chronic heart failure (ejection fraction 22%) and interstitial lung fibrosis. Given the patient's advanced age and the significant impact of urinary symptoms and recurrent gross haematuria on his quality of life, a shared decision was reached to proceed for HoLEP. After the surgery, the patient was diagnosed with clinically significant prostate cancer, but he opted for AS. His metastatic workup was negative, and nadir PSA was 0.7 ng/dL. This management approach reflects a complex interplay of factors, including the patient's advanced age, associated comorbidities, the desire to mitigate bothersome urinary symptoms, and a cautious approach to the management of prostate cancer. It is essential to acknowledge that each patient's case must be assessed individually, with careful consideration of the risks, benefits, and patient preferences, in order to determine the most appropriate management strategy.

We conducted a retrospective study at a single institution, and therefore, it has inherent limitations. We could not confirm any preoperative demographic or biochemical factors that were associated with high likelihood of detecting iPCa as found by other investigators. This might be explained by our protocol of aggressive preoperative screening for prostate cancer with almost 37.4% of all patients undergoing HoLEP having at least one negative preoperative prostate biopsy. Nonetheless, logistic regression analysis focussing on the MRI findings could not be incorporated in our study due to the decreased number of patients who underwent MRI prior to prostate biopsy. However, our study would help in preoperative counselling of patients about these sensitive issues and would also help to reassure patients that diagnosis of iPCa is unlikely to negatively impact voiding improvement after HoLEP. Regarding the significant association between hypertension and GG 1 prostate cancer, it is crucial to emphasize that while prostate cancer is influenced by numerous factors, we do not have a cause‐and‐effect relationship to elucidate this finding. Moreover, we believe that incidentally detecting high‐risk prostate cancer after HoLEP reflects the limitations in the current diagnostic workup causing distress for the patient. There is a need for large prospective multi‐institutional studies aiming to develop a predictive model for identifying the likelihood iPCa at BPH procedures. This way, these studies would include various patient parameters that could assist urologists and patients in risk stratification and personalized decision‐making.

## 5 CONCLUSIONS

Our study sheds light on the potential of detecting iPCa during HoLEP procedures, with a prevalence rate of 9.6%. Prior negative prostate biopsies, prostate size, or PSA level were not associated with increased risk of iPCa. Preoperative MRI and prostate biopsy failed to diagnose 20% of patients with clinically significant prostate cancer, and all patients with PIRADS 4 or 5 lesions in spite of negative fusion biopsy were diagnosed with clinically significant prostate cancer. It is imperative for healthcare providers to inform patients about this possibility when considering surgical management for BPH. Nevertheless, the majority of cases was low‐risk prostate cancer and was managed through AS.

## AUTHOR CONTRIBUTIONS

**Joao G. Porto**: Data collection; writing/editing manuscript. **Ruben Blachman‐Braun**: Project development; data analysis; writing/editing manuscript. **Tarek Ajami**: Project development; writing/editing manuscript. **Mohamadhusni Zarli**: Data collection. **Ryan Chen**: Data collection. **Thiago Furtado**: Writing/editing manuscript. **Robert Marcovich**: Project development; writing/editing manuscript. **Dipen J. Parekh**: Project development; writing/editing manuscript. **Hemendra N. Shah**: Project development; writing/editing manuscript.

## CONFLICT OF INTEREST STATEMENT

The authors declare no conflict of interest.
